# Incidence Patterns and Outcomes for Hodgkin Lymphoma Patients in the United States

**DOI:** 10.1155/2011/725219

**Published:** 2010-12-16

**Authors:** Pareen Shenoy, Alison Maggioncalda, Neha Malik, Christopher R. Flowers

**Affiliations:** Winship Cancer Institute, Emory University, 1365 Clifton Road, N.E. Building B, Suite 4302, Atlanta, GA 30322, USA

## Abstract

Hodgkin lymphoma (HL) demonstrates heterogenous histologic findings, clinical presentation, and outcomes. Using the United States Surveillance, Epidemiology, and End Results (SEER) data we examined relationships between patient characteristics, clinical features at diagnosis, and survival in HL patients. From 2000 to 2007, 16,710 cases were recorded in 17 SEER registries. Blacks and Asians had low incidence (black/white incidence rate ratio (IRR) 0.86, *P* < .01; Asian/white IRR 0.43, *P* < .01). The bimodal pattern of incidence was less prominent for black males. Asians and Blacks presented at a mean age of 38 years compared to 42 years for Whites (*P* < .001). Race was a predictor for survival with HR of 1.19 (95% CI 1.11–1.28) for Blacks. Age was the most important predictor of survival (HR for patients ≥45 years 5.08, 95% CI 4.86–5.31). These current patterns for presentation and outcomes of HL help to delineate key populations in order to explore risk factors for HL and strategies to improve treatment outcomes.

## 1. Introduction

Hodgkin lymphoma (HL) is a cancer of the lymphatic system commonly characterized by the presence of large malignant lymphoid cells, Reed-Sternberg cells, although malignant cells typically account for less than 1% of cells in the affected tissue [1]. The variable quantity and quality of the malignant cells and the host response make the histologic findings in HL heterogenous [2]. In the United States (US), an estimated 8,490 new cases were diagnosed with HL while HL was accountable for 1,320 deaths in 2010 [3]. In the 1960s, the 5-year survival rate for HL was less than 10% [4]. With breakthroughs in combination chemotherapy regimens, the reported 5-year survival for patients with HL during the years 2000–2004 was 85.2% [5]. However, relatively little is known regarding the clinical and demographic factors that influence the patterns of presentation and outcomes for HL in a population-based setting in the US.

Recent epidemiological studies have found independent associations between age, gender, race, geographic location, and incidence of HL [2]. For example, Asians have been shown to have a dramatically lower HL incidence than other races, but even among US Asians there are significant incidence rate differences between US-born Asians versus native Asians [6]. The incidence variation by age, geographic location, social class, and time suggests an etiologic role for infectious agents, such as Epstein-Barr virus (EBV), while aggregation in families and persons with specific human leukocyte antigen (HLA) types indicates genetic susceptibility [2, 7]. These and other factors influencing HL incidence and outcomes are reviewed elsewhere in this journal [7]. To examine the relationships between demographic patient characteristics, clinical features at diagnosis, and survival outcomes in a national cohort of patients with HL, we performed a retrospective analysis of HL cases diagnosed from 1973 to 2007 reported to population-based cancer registries.

## 2. Methods

### 2.1. Data Sources

We obtained population-based cancer incidence data from the United States Surveillance, Epidemiology, and End Results (SEER) Program. This database has compiled incidence data since 1973 from nine population-based registries (Connecticut, Hawaii, Iowa, New Mexico, Utah, Detroit, San Francisco/Oakland, Seattle/Puget Sound, and Atlanta), accounting for 10% of the United States population [8]. The SEER 9 registries population is comparable to the general population except containing a higher proportion of urban areas and foreign-born persons [9]. In 1992, SEER expanded to include two additional metropolitan areas (Los Angeles County and San Jose/Monterey, California), Rural Georgia, and the Alaska Native Tumor Registry, together accounting for approximately 14% of the United States population [10, 11]. Later in 2000, SEER included Greater California, Kentucky, Louisiana, and New Jersey registries to account for 26% of the US population [12]. For the analyses of incidence rates, we used the SEER17 data compiled of cases diagnosed from January 1, 2000 to December 31, 2007 [12]. Given its longer duration of followup, for the survival analysis, we used the SEER9 data compiled of cases diagnosed from January 1, 1973 to December 31, 2007 [7]. 

### 2.2. Study Cohort

The classification of lymphoid neoplasms has undergone several updates since the 1940s. Lymphomas diagnosed from 1973 through 1977 were classified according to the Manual of Tumor Nomenclature and Coding [13]. Then in 1978, the International Classification of Diseases for Oncology (ICD-O) [14] was adopted to code all cancers registered by the SEER Program. In 1992, the SEER Program adopted the ICD-O, 2nd Edition (ICD-O-2) to code lymphomas [15]. In 2001, the World Health Organization (WHO) classification was introduced building on the Revised European-American Lymphoma classification and the French-American-British classification [16, 17] and updated in 2008 [18]. Recently the United States cancer registries adopted the ICD-O-3 with the ability to convert cases coded in ICD-O-2 to ICD-O-3 [19] making it feasible to assess incidence patterns and trends for lymphoid neoplasms according to the internationally recognized WHO classification [20]. 

**Figure 1:**
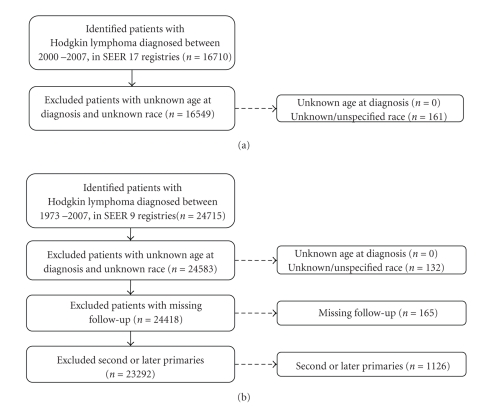
Selection of study cohort. These figures provide an overview of the study cohort with reasons for inclusion/exclusion through the selection process. (a) Selection of the cases included in the incidence analyses. (b) Selection of the cases included in the survival analyses.

We identified and categorized HL cases using the third edition of the International Classification of Diseases for Oncology (ICD-O-3) [21] into classical HL (CHL) and nodular lymphocyte predominant HL (NLP, ICD-O-3 9659). CHL cases were further divided into the following subtypes: lymphocyte-rich (LR, 9651), mixed cellularity (MC, 9652), lymphocyte-depleted (LD, 9653–9655), nodular sclerosis (NS, 9663–9667), and not-otherwise-specified (NOS, 9650–9651) (Table 1). This corresponds with the InterLymph clustering of the WHO classification of lymphoid malignancies into categories designed for lymphoma epidemiological research [22]. All data refer to the incidence of neoplasms with malignant behavior. The selection of HL cases for analysis of incidence and survival is depicted in Figures 1(a) and 1(b), respectively. Cases with unknown or unspecified race were excluded from the analysis. Cases with known race were grouped into white, black, American Indians or Alaska Natives (AI/ANs), and Asians or Pacific Islanders (A/PIs). The SEER program records race as assigned by the North American Association of Central Cancer Registries [23]. Race and ethnicity were defined by specific physical, hereditary, and cultural traditions or origins, not necessarily by birthplace, place of residence, or citizenship. Examination of Hispanic ethnicity was beyond the scope of this study because ethnicity was not recorded reliably in the SEER registries during this time period [24]. 

 SEER data were used to calculate survival time using the date of diagnosis and one of the following: date of death, date last known to be alive, or date of the study cutoff (December 31, 2007). For survival analyses, patients whose disease status was based on a death certificate or autopsy only, patients with second or later primaries, and patients who were not actively followed were excluded, resulting in a cohort of (23,292) cases. To evaluate the impact of modern therapies on survival, we divided patients into eras by year of diagnosis: 1973–1979, 1980–1989, 1990–1999, and 2000–2007.

### 2.3. Calculation and Presentation of Rates

Incidence rates were expressed as new cases per 100,000 person-years and age-adjusted to the 2000 U.S. standard population [25]. Incidence rates were compared by sex and race using incidence rate ratios (IRRs) and 95% confidence intervals (95% CIs). Differences in baseline characteristics at diagnosis across racial groups were analyzed using two-sided *t*-tests and chi-square tests.

Two-year and five-year relative survival rates (RSRs) were calculated by actuarial methods, where relative survival is defined as the ratio of the proportion of observed survivors in a cohort of cancer patients to the proportion of expected survivors in a comparable set of cancer-free individuals, thus representing survival in the absence of other causes of death. A *Z*-test was used to test the equivalence of RSRs [26]. Survival curves were constructed using the Kaplan-Meier method and compared with the log-rank test. Univariate and multiple variable Cox proportional hazards models were used to examine the covariates age at diagnosis, gender, race, stage of disease, presence of extranodal disease, and presence of B-symptoms as predictors of mortality.

A level of significance (*α*) of  .05 was considered statistically significant. All statistics were computed using the National Cancer Institute SEER*Stat software, version 6.5.2. (http://www.seer.cancer.gov/seerstat/), SAS software, version 9.0 (SAS Institute Inc. Copyright ©2002), and STATA 9.2 (StataCorp LP. Copyright ©1855–2006).

## 3. Results

### 3.1. Incidence of HL

During the period from 2000 to 2007, 16,710 cases of HL were diagnosed and reported to the 17 SEER registries. Of these, 161 cases with unknown/unspecified race were excluded resulting in a study cohort of 16,549 cases: 14,076 whites, 1,693 blacks, 58 AI/AN, and 722 A/PI. The study populations for the incidence and survival analyses are shown in Figure 1. The age-adjusted incidence rate for HL was 2.74 (95% CI 2.69–2.78), NLP was 0.11 (95% CI 0.1–0.12), and for CHL was 2.63 (95% CI 2.59–2.67). The age-adjusted incidence rates for the CHL subtypes LR, MC, LD, NS, and CHLNOS were 0.08 (95% CI 0.07–0.09), 0.37 (95% CI 0.35–0.39), 0.04 (95% CI 0.03–0.04), 1.63 (95% CI 1.59–1.66), and 0.51 (95% CI 0.49–0.53), respectively (Table 1).

### 3.2. Incidence Rates by Sex and Race

Males had significantly higher incidence rates than females for all HL subtypes. The male-to-female (M/F) IRR was 1.27 (95% CI 1.23–1.31) for all HL combined and greatest for NLP HL (2.45, 95% CI 2.1–2.9). Male predominance among HL was most pronounced among A/PI (M/F IRR 1.35, 95% CI 1.2–1.6). Although the overall incidence rates for HL were significantly lower for black patients than white patients (black-to-white IRR 0.86, *P* < .01), black patients had higher incidence rates compared to white patients for NLP HL (black-to-white IRR 1.9, *P* < .01; Table 1). The difference in incidence between whites and Asians was even more prominent (Asian-to-white IRR 0.43, *P* < .01).

### 3.3. Age-Specific Incidence Rates

**Figure 2:**
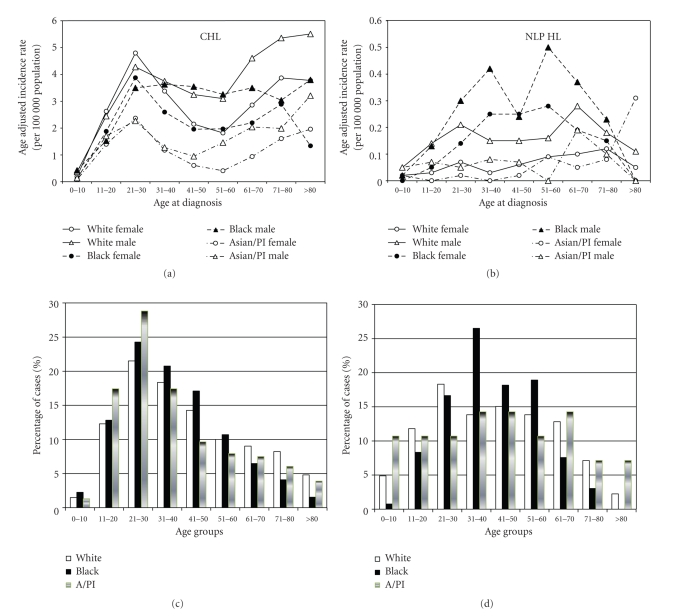
Age distribution of HL by race. (a) Plots are the race- and gender-specific age-adjusted incidence rates of CHL by age at diagnosis. (b) Plots are the race- and gender-specific age-adjusted incidence rates of NLP HL by age at diagnosis. (c) Plots are the age distribution of CHL patients. Horizontal axis represents the grouping of age at diagnosis. Vertical axis represents the proportion of patients in each age group for that particular race (White, Black, and Asian/Pacific Islander). (d) Plots are the age distribution of NLP HL patients. Horizontal axis represents the grouping of age at diagnosis. Vertical axis represents the proportion of patients in each age group for that particular race (White, Black, and Asian/Pacific Islander).

The age distribution of CHL across gender and race differed. White patients demonstrated a bimodal distribution with prominent peaks at age 21–30 and >70 years, although the second peak for white females was less prominent (Figure 2(a)). This bimodal age distribution pattern was seen in all groups except for black males with CHL. For NLP HL, however, the only obvious bimodal incidence pattern was seen for black males, with peak incidence rates at 31–40 years and 51–60 years.

### 3.4. Baseline Characteristics at Diagnosis

The baseline characteristics at diagnosis by race are compared in Table 2. The mean age at diagnosis for black patients and Asian/PI was younger than that for white patients (both *P* < .001; Table 2). Blacks had a higher percentage of cases with an extranodal disease (27% versus 23%, *P* < .001), but no significant difference in the percentage of patients with B symptoms (36% versus 35%, *P* = .058) compared to white patients. Compared to white patients, black patients had a higher percentage of cases diagnosed with stage III/IV disease (43% versus 35%, *P* < .001). Asian/PI patients also had a slightly higher percentage of cases with stage III/IV HL (38% versus 35%, *P* < .001). Information on stage, extranodal disease, and B-symptoms was missing in 5.7%, 5.7%, and 24% of cases, respectively.

### 3.5. Survival Analysis

The 2-year and 5-year RSRs for the entire population of patients HL were 87% and 80%. The 2-year RSRs for CHL subtypes NS; LR; MC; LD; CHL, NOS; NLP HL were 92%, 93%, 81%, 49%, 75%, and 97%, respectively. The 5-year RSRs for CHL subtypes NS; LR; MC; LD; CHL, NOS; NLP HL were 86%, 87%, 73%, 43%, 68%, and 94%, respectively. Female patients had better RSRs than males (2-year RSR: 88% versus 86%, 5-year RSR: 83% versus 78%, both *P* < .001). White, black, and A/PI patients had similar survival rates (2-year RSR: 87%, 85%, and 84%, resp., 5-year RSR: 81%, 77%, and 77%, resp., *P* < .01). However, race was a significant predictor of survival in Cox regression models (Table 3). In multiple variable Cox regression models, the most significant predictors of survival were age ≥45 (HR 4.82, 95% CI 4.40–5.29), B symptoms (HR 1.82, 95% CI 1.66–2.00), black race (HR 1.54, 95% CI 1.35–1.76), stage IV disease (HR 1.29, 95% CI 1.05–1.59), and male gender (HR 1.17, 95% CI 1.07–1.28; Table 3). Multiple variable Cox regression models demonstrated that when compared to NS HL, MC HL, LD HL, and CHL, NOS had worse survival with HR of 1.31 (95% CI 1.17–1.46), 2.02 (95% CI 1.58–2.57), and 1.65 (95% CI 1.46–1.86), respectively (Table 3).

**Figure 3:**
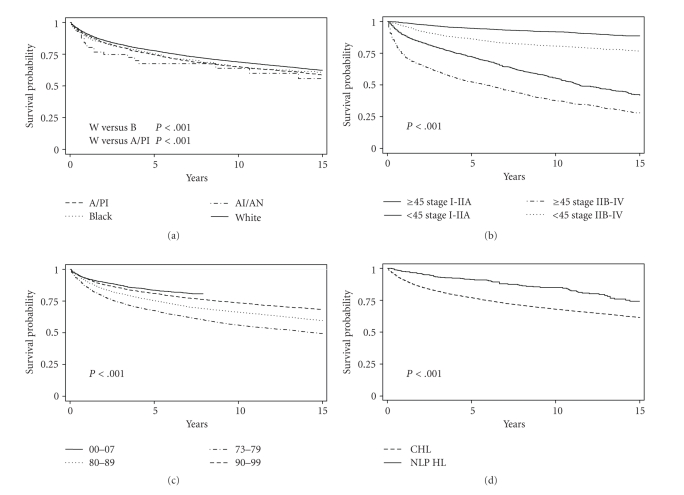
Kaplan-Meier Survival Curves of HL Patients. (a) Survival curves by race. (b) Survival curves by age at diagnosis (<45 versus ≥45 years) and stage (Stage I-IIA versus Stage IIB-IV). (c) Survival curves by era of diagnosis. (d) Survival curves for CHL and NLP HL.

There was a statistically significant improvement in survival across consecutive diagnostic eras (log-rank test *P* < .001, Figure 3(c)) with similar trends observed for each racial group. The improvement in survival across the diagnostic eras was also seen uniformly in all subtypes. The 5-year RSR for NLP HL was 71.5% for patients diagnosed in the years 1973–79; 91.8% (1980–89); 91.5% (1990–99); 98.9% (2000–07). Those for NS HL were 80.1% (1973–79); 84.2% (1980–89); 88% (1990–99); 89.9% (2000–07). Kaplan-Meier survival curves also show a significant difference in survival by age at diagnosis, with patients who were ≥45 years having a worse outcome (log-rank test *P* < .001). Across histologic subtypes, survival for patients with NLP HL was better than that for patients with CHL (log-rank test *P* < .001; Figure 3(d)). Although stage of disease did affect survival outcomes, age ≥45 years had a greater impact on survival (Figure 3(b)).

## 4. Discussion

Our study examines incidence, presentation, and survival of HL with respect to previously assessed risk factors: age, gender, race, stage, and histologic subtype. Although these risk factors have been examined in prior studies, the majority of these studies focused on one factor independent of the others. Using the most recent national SEER data, we analyzed the relationships between patient demographics, presentation of disease, and overall survival in an attempt to identify which risk factors had the most influence on incidence and survival for HL.

Lack of central pathology review is a limitation of this and all other SEER studies. In our study cohort, patients included were diagnosed with HL based on local healthcare system standards. Another possible limitation of this analysis is the use of cases classified according to ICD-O-2, prior to the introduction of the revised WHO classification in 2001. Clarke et al. studied the reliability of computer-converted ICD-O-2 codes to ICD-O-3 codes in SEER and found that the agreement for classical HL was 95% while that for nodular HL was 44% [19, 27]. 

In the current analyses, we categorize racial groups into white, black, AI/AN, and A/PI. It should be noted, however, that clumping all Asians together might not be meaningful for detailed epidemiologic studies since it lumps together genetically distinct and heterogenous populations of patients who would likely have different genetic, dietary, and possibly environmental risks for lymphoma. Although the SEER data provide detailed information on race at the case level, it classifies race into white, black, AI/AN, and A/PI at the population level; thereby precluding further analyses of racial subgroups with this dataset. Another limitation of the SEER data is the absence of information on the HIV status of the patients. This precludes that analysis of incidence of HIV-positive patients across racial groups.

### 4.1. Incidence

As expected, HL incidence was higher in males than females and higher in whites than other races. These data also address the debate over the existence of a bimodal incidence pattern for HL with current population-based data. As Figure 2 displays, there are clear CHL incidence peaks for young adults and individuals ≥70 years of age for both genders and all racial groups except for black males, where the peaks are not nearly as prominent. While a bimodal incidence pattern of HL is largely debated, these findings are consistent with several other studies [28–30]. 

In terms of race, both blacks and Asian/Pacific Islanders had lower incidence rates than whites for CHL. Asians had significantly lower incidence rates for virtually all HL subtypes. Internationally, Asians have lower HL incidence than other races. According to Glaser and Hsu, consistently low incidence rates of HL in the Asian population as a whole suggest genetic resistance, possibly related to HLA type, and differences between US-born and native Asian groups suggest environmental influences [6]. HLA type has been shown to affect EBV-related HL incidence, and EBV-positive HL is most common in the Asian population [31, 32]. Though its impact seems dependent on age, early microbiome exposure has been linked to having a protective effect against HL [33, 34]. Racial differences in early exposure to viral pathogens and microbiomes may influence these differences in the incidence of HL and the patterns of HL presentation discussed below. For most HL subtypes, black race was associated with lower HL incidence rates. However, for NLP HL, blacks had a significantly higher incidence rate than whites. This is somewhat surprising given that NLP HL has a clinical behavior similar to indolent non-Hodgkin lymphomas (NHLs), and black Americans also have lower incidence of NHL [35]. Further studies investigating the etiology of racial differences in incidence patterns for HL using admixture mapping will help to elucidate genetic susceptibility and gene-environment interaction associated with the incidence of HL and HL subtypes.

### 4.2. Presentation

We found significant differences in the clinical features at presentation across the racial groups studied. These differences may contribute to racial differences in survival since age of onset, stage, and B-symptoms at diagnosis are known prognostic factors for HL. Not only did blacks and Asian/PI present at a lower age than whites (38 versus 42 years), but both groups also had a higher percentage of patients with stage III/IV disease. Additionally, blacks had a higher percentage of patients presenting with an extranodal disease. One possible explanation for these differences in presentation could be racial differences in the prevalence of human immunodeficiency virus (HIV) among individuals with HL. HIV-positive HL patients present at a more advanced stage with associated extranodal involvement and B symptoms [36]. Biggar et al. found that individuals with HIV/AIDS were also more likely than the general population to be diagnosed with HL. AIDS cases with moderate immunosuppression (225–249 CD4 cells/*μ*L at onset) were estimated to have a 15-fold increased risk of HL [37]. In Glaser's HIV related HL study, 21.3% of blacks in the study were HIV positive, whereas only 13% of whites and 2.3% of Asians were HIV-positive [36]. On a national scale, blacks are significantly overrepresented in HIV/AIDS incidence [38]. Patients of nonwhite race were diagnosed earlier and with a higher level of disease progression. Recent epidemiological studies in NHL also found significant differences between blacks and whites in the age of presentation and racial differences in overall survival [35, 39–41].

### 4.3. Survival

Age, gender, presenting symptoms, stage, and race were all predictors of survival according to our multiple variable Cox regression. The international prognostic score for advance stage HL identified seven factors with similar independent prognostic effects: serum albumin level <4 g per deciliter, hemoglobin level <10.5 g per deciliter, male sex, an age of ≥45 years, stage IV disease, white-cell count >15,000 per cubic millimeter, and lymphocyte count <600 per cubic millimeter, <8% of the white-cell count, or both [42]. In this population-based analysis, age of 45 or above, stage IV disease, and male gender remained significant predictors of HL survival for patients with advanced stage disease (data not shown) and all patients. The remaining components of the prognostic score are laboratory based and therefore not able to be analyzed with SEER. 

Our data showed that an age of 45 or older had an overwhelming association with mortality (HR 5.25). The next highest predictor was the presence of B symptoms (HR 1.90), followed by black race (HR 1.54). Only after these factors did stage IV disease (HR 1.39) and male gender (1.19) come into play. Age was significantly more predictive of survival than stage (Figure 3(b)). Interestingly enough, both race and B-symptoms at presentation also were significant predictors of survival in multiple variable regression models. Determining whether the impact of race and B-symptoms are confounded by differences in the prevalence of HIV or EBV in these groups needs to be addressed with additional studies, since both viruses can influence the incidence and survival of HL [34, 36]. 

As mentioned previously, black individuals also have a higher incidence than whites of NLP HL. Using NS as a reference, MC, LD, and CHL NOS subtype diagnoses were predictive of worse 5-year survival (Table 3). Cox regression models showed that NLP was the only subtype that predicted for better survival than NS (Table 3), and the overall survival curve (Figure 3(d)) suggests that while NLP HL may have worse initial outcomes, long-term survival is better for NLP HL patients than for CHL patients. It should however be noted while interpreting these survival analyses that there might be potential misdiagnosis of NLP HL as T-cell-rich B-cell lymphoma or follicular lymphoma. Additional HL subtype-specific studies are needed to investigate factors that influence outcomes particularly with the future development of individualized treatment strategies for HL subtypes. 

Finally, the survival curves in Figure 3(c) demonstrate that the survival probability continues to improve for patients with HL across eras of diagnosis. This is particularly interesting since in the 1980s the combination chemotherapy regimen of doxorubicin, bleomycin, vinblastine, and dacarbazine (ABVD) supplanted previous treatment strategies such as radiation therapy alone and nitrogen mustard, vincristine, procarbazine, and prednisone (MOPP) and has remained the standard of care for HL ever since [43–49]. However, despite the commonplace use of this regimen across most of the eras studied, HL survival has serially improved. We speculate that an increase in the access to care, improvement of diagnostic tools such as computed tomography and positron emission tomography scans, and efficacy of supportive care may be responsible for these increases in survival. A Spanish study found that patients treated before 1980 were 3.5 times more likely to die of infection and 7.1 times more likely to die of toxicity than those treated after 1980 [50]. Another reason for the improved survival in recent years could be due to improved diagnostic accuracy and elimination of the misdiagnosis of HL cases as NHL in the earlier years. The greatest additional improvements in HL survival are likely to come from identifying poor risk subgroups and developing management approaches specific for these populations that do not benefit from standard therapy. Studies such as this one help to identify subgroups (black patients, patients 45 years or older, and patients with stage IV disease) where specific interventions such as improving access to care, adding biological therapies, and intensifying initial therapy may be explored to improve survival for these poor-risk populations.

## Figures and Tables

**Table 1:** Age-adjusted incidence rates of Hodgkin lymphomas by subtype, 17 SEER registries, 2000–2007.

				Incidence rate ratio*			
				Male		Female	
Hodgkin lymphoma subtype	ICD-O-3 codes	Count	Age-adjusted incidence rate (CI)	Black	Asian/PI	Black	Asian/PI
*Classical Hodgkin lymphoma*	9650–9655, 9661–9667	15,895	2.63 (2.59–2.67)	0.86 (0.79–0.93)	0.45 (0.40–0.49)	0.79 (0.73–0.85)	0.41 (0.36–0.46)
Lymphocyte-rich	9651	501	0.08 (0.07–0.09)	0.96 (0.65–1.39)	0.57 (0.32–0.93)	1.56 (0.99–2.37)	0.61 (0.27–1.19)
Mixed cellularity	9652	2,197	0.39 (0.35–0.39)	0.99 (0.83–1.19)	0.45 (0.34–0.59)	0.89 (0.69–1.13)	0.43 (0.29–0.61)
Lymphocyte-depleted	9653–9655	225	0.04 (0.03–0.04)	0.78 (0.40–1.41)	0.62 (0.25–1.28)	0.76 (0.29–1.65)	0.28 (0.03–1.01)
Nodular sclerosis	9663–9667	9,927	1.63 (1.59–1.66)	0.74 (0.66–0.82)	0.43 (0.37–0.49)	0.72 (0.65–0.79)	0.41 (0.35–0.47)
Classical Hodgkin lymphoma, NOS	9650, 9661, 9662	3,045	0.51 (0.49–0.53)	1.08 (0.92–1.26)	0.45 (0.34–0.57)	0.92 (0.76–1.11)	0.37 (0.27–0.49)
*Nodular lymphocyte predominant Hodgkin lymphoma*	9659	654	0.11 (0.10–0.12)	1.66 (1.27–2.14)	0.44 (0.25–0.71)	2.84 (2.02–3.95)	0.67 (0.31–1.28)
* Hodgkin lymphoma*	9650–9655, 9659, 9661–9667	16,549	2.74 (2.69–2.78)	0.89 (0.83–0.96)	0.45 (0.40–0.49)	0.83 (0.77–0.89)	0.41 (0.37–0.46)

Abbreviations: ICD-O: international classification of diseases for oncology; CI: 95% confidence interval; PI: Pacific islanders; NOS: not otherwise specified.

*Ratio of age-adjusted incidence rates Black/White and Asian/White.

**Table 2:** Racial differences in the presentation of Hodgkin lymphoma.

Characteristic	White		Black		A/PI		*P*-value	
	Count	%	Count	%	Count	%	W versus B	W versus A/PI
*n*	14,076		1,693		722			
Age, years								
Mean	42		38		38		<.001	<.001
Interquartile Range	26–57		25–49		23–51			
<45	8,472	60.19	1,148	67.81	493	68.28	<.001	<.001
≥45	5,604	39.81	545	32.19	229	31.72		
Male sex	7,711	54.78	913	53.93	399	55.26	.51	.80
Stage								
I	2,717	19.30	360	21.26	95	13.16	<.001	<.001
II	5,555	39.46	527	31.13	327	45.29		
III	2,639	18.74	365	21.56	133	18.42		
IV	2,349	16.69	356	21.03	128	17.73		
Unknown	816	5.80	85	5.02	39	5.40		
Extranodal disease								
Yes	3,170	22.52	455	26.88	179	24.79	<.001	.17
No	10,090	71.68	1,153	68.10	504	69.81		
Unknown	816	5.80	85	5.02	39	5.40		
B symptoms								
Yes	4,988	35.44	608	35.91	305	42.24	.06	.60
No	5,783	41.08	629	37.15	275	38.09		
Unknown	3,305	23.48	456	26.93	142	19.67		

Note: for each variable, cases with unknown information were excluded in calculating the *P*-value.

Abbreviations: W: whites; B: blacks; O: others.

**Table 3:** Cox regression models of predictors mortality among Hodgkin lymphoma patients.

Factors	Univariate model			Multiple variable model		
	Hazard ratio	95% CI		Hazard ratio	95% CI	
Age, years						
<45	1.00			1.00		
≥45	5.08	4.86	5.31	4.82	4.40	5.29
Gender						
Female	1.00			1.00		
Male	1.32	1.27	1.38	1.17	1.07	1.28
Race						
White	1.00			1.00		
Black	1.19	1.11	1.28	1.54	1.35	1.76
Asian/PI	1.11	0.97	1.26	1.09	0.86	1.37
AI/AN	1.32	0.87	2.01	0.83	0.39	1.74
Disease stage						
I/III	1.00			1.00		
IV	2.45	2.26	2.65	1.29	1.05	1.59
Extranodal disease						
No	1.00			1.00		
Yes	2.17	2.01	2.34	1.10	0.91	1.34
B symptoms						
No	1.00			1.00		
Yes	2.20	2.01	2.40	1.82	1.66	2.00
HL subtype						
NS HL	1.00			1.00		
LR HL	1.29	1.15	1.44	1.10	0.86	1.40
MC HL	1.99	1.89	2.09	1.31	1.17	1.46
LD HL	4.08	3.71	4.48	2.02	1.58	2.57
CHL, NOS	2.17	2.04	2.30	1.65	1.46	1.86
NLP HL	0.74	0.60	0.91	0.71	0.52	0.98
